# The Regulation and Secretion of Glucagon in Response to Nutrient Composition: Unraveling Their Intricate Mechanisms

**DOI:** 10.3390/nu15183913

**Published:** 2023-09-08

**Authors:** Jiudan Zhang, Yang Zheng, Lisa Martens, Andreas F. H. Pfeiffer

**Affiliations:** 1 The First Affiliated Hospital of Zhejiang Chinese Medical University, Hangzhou 310053, China; jiudan.zhang@charite.de; 2 Department of Endocrinology, Diabetes and Nutrition, Charité-Universitätsmedizin Berlin, Corporate Member of Freie Universität Berlin, Humboldt-Universität zu Berlin, and Berlin Institute of Health, 10117 Berlin, Germany; lisa.martens39@gmail.com (L.M.); andreas.pfeiffer@charite.de (A.F.H.P.); 3 Nutritional Science, University of Potsdam, 14469 Potsdam, Germany

**Keywords:** glucagon, glucose, lipid, amino acid, hyperglucagonemia

## Abstract

Glucagon was initially regarded as a hyperglycemic substance; however, recent research has revealed its broader role in metabolism, encompassing effects on glucose, amino acids (AAs), and lipid metabolism. Notably, the interplay of glucagon with nutrient intake, particularly of AAs, and non-nutrient components is central to its secretion. Fasting and postprandial hyperglucagonemia have long been linked to the development and progression of type 2 diabetes (T2DM). However, recent studies have brought to light the positive impact of glucagon agonists on lipid metabolism and energy homeostasis. This review explores the multifaceted actions of glucagon, focusing on its regulation, signaling pathways, and effects on glucose, AAs, and lipid metabolism. The interplay between glucagon and other hormones, including insulin and incretins, is examined to provide a mechanistic understanding of its functions. Notably, the liver–α-cell axis, which involves glucagon and amino acids, emerges as a critical aspect of metabolic regulation. The dysregulation of glucagon secretion and its impact on conditions such as T2DM are discussed. The review highlights the potential therapeutic applications of targeting the glucagon pathway in the treatment of metabolic disorders.

## 1. Introduction

Glucagon, a 29-amino acid peptide, was discovered in 1921 [1] and was described as a hyperglycemia substance due to contaminants in pancreatic extracts [2] in 1923. In 1948, it was established that glucagon is released from pancreatic α-cells and later, to a lesser extent, from brainstem neurons [3,4]. It is widely recognized that fasting and postprandial hyperglucagonemia play a crucial role in both the development and progression of type 2 diabetes (T2DM). However, researchers recently reshaped the role of glucagon in metabolism, confirming that the biology of glucagon is more comprehensive and extends beyond hepatic hyperglycemic actions to exert effects on glucose, amino acids (AAs), and lipid metabolism. 

The hyperglycemic effect in T2DM is undisputedly present, as demonstrated by glucagon receptor antagonists in humans that, however, induced hepatic side effects [5,6,7]. The positive effects of glucagon agonists on lipid metabolism, energy homeostasis, and the reduction of liver fat have been emphasized with the development of glucagon/GLP-1 co-agonists as well as GLP-1/GIP/glucagon triple agonists, which are currently in clinical development/trials [8,9,10].

Nutrients, especially amino acids (AAs), and non-nutrient components stimulate glucagon secretion directly through sensory transporters and receptors or indirectly through their effects on cellular metabolism. Indeed, increasing protein intake, and thereby glucagon release, has shown positive effects in studies with orally treated T2DM patients [11,12]. We therefore review studies addressing the role of glucagon regulation by food intake. We describe the complex interplay of glucagon with glucose, protein/amino acid and lipid/fatty acid metabolism as well as the secretion of insulin and other hormones to provide a mechanistic background. Our hypothesis is that the powerful stimulation of glucagon by AAs and other food components may be exploited in the treatment of T2DM.

## 2. Glucagon Actions and Regulation

In the decades following its discovery, glucagon was viewed primarily as the counter-regulatory hormone of insulin, governing glucose homeostasis [13,14]. Nowadays, glucagon is considered as a pleiotropic hormone whose metabolic actions include insulin secretion [15], the regulation of lipid and AA metabolism, increasing energy expenditure, the modulation of food intake and satiety, and the facilitation of weight loss in both animals and humans [16,17,18]. Furthermore, glucagon’s influence extends to the regulation of bile acid metabolism, encompassing processes such as bile acid synthesis, uptake, and efflux [19].

**Figure 1:**
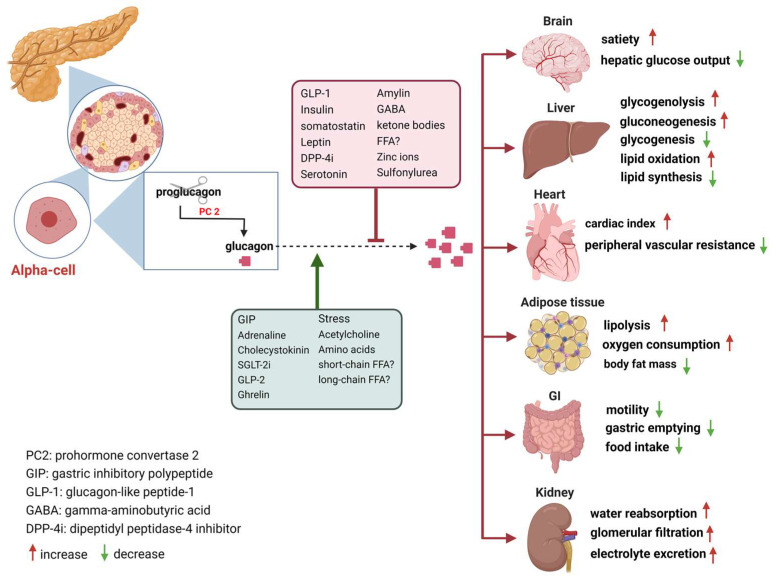
Glucagon receptors on multiple organs and the stimulation/inhibition of glucagon release. (This graph was generated with www.biorender.de).

The secretion of glucagon by α-cells is primarily associated with occurrences of hypoglycemia, acting as a safeguard against low blood sugar levels. Additionally, it is also stimulated by elevated circulating levels of AAs and fatty acids, as well as in response to adrenergic stimulation and circulating incretins [20]. Notably, established evidence indicates that β-cell-derived secretory products, including insulin, zinc and gamma-aminobutyric acid (GABA), inhibit glucagon secretion [21]. Meanwhile, it is potently suppressed by somatostatin, GLP-1, amylin, leptin, fatty acids, and ketone bodies and stimulated by GIP and vagal stimulation. Meier et al. demonstrated that GLP-2 also stimulates glucagon secretion in healthy subjects [22] (Figure 1). And some medications such as furosemide or acetylsalicylic acid may influence prostaglandin (PG) synthesis, mainly PGE, which in turn controls glucagon release. In this regard, details of glucagon receptor function are helpful to understand its link to the metabolic effects of glucagon.

Additionally, it had been reported early in 1982 that a pulsatile secretion pattern for both insulin and glucagon exists in healthy individuals, which might be stimulated by alpha–beta intercellular connections [23]. The secretion of these hormones in pulses allows for precise and fine-tuned control over blood glucose levels, preventing hypoglycemia/hyperglycemia. However, in individuals with prediabetes or T2DM, this delicate balance can become disrupted or lost [24,25]. It is conceivable that postprandial hyperglucagonemia in T2DM individuals is due to impaired postprandial insulin secretion, as T2DM is characterized by a significant impairment in postprandial insulin secretion [26]. The disruption in the pulsatile secretion of both hormones can disturb glucose hemostasis and may lead to the development of hyperglycemia and insulin resistance. The exact mechanisms behind this phenomenon are complex and not fully understood, but they likely involve defects in the signaling pathways and communication between different types of islet cells in the pancreas. 

### How Are GCGRs Regulated?

Glucagon receptors (GCGRs) belong to the class B of seven-transmembrane (7TM) protein receptors known to activate adenylyl cyclase through Gαs-coupled proteins, which is accompanied by an increase in cellular cyclic AMP levels and activation of protein kinase A (PKA) [27,28]. Recently, GCGRs were also shown to activate the IP3 pathway via Gq and the activation of the inositol triphosphate receptor 1 (INSP3R1) in liver cells [29]. GCGRs are highly expressed in the liver as well as in multiple extrahepatic tissues (Figure 1), which play an essential role in glucose, AA, lipid, and energy metabolism [21,30,31]. 

Delving into the regulation of GCGRs, pivotal insights emerged from research in 1996 that linked diminished glucagon binding to reduced lipolysis, suggesting that downregulation of cell surface GCGRs drives the desensitization of glucagon’s capacity to stimulate lipolysis in adipocytes [32]. It has been reported that dihydroxy bile acids like chenodeoxycholic acid (CDCA) induce GCGR desensitization [33]. A study in 2006 demonstrated that CDCA stimulated the phosphorylation and heterologous desensitization of the GCGRs through protein kinase C (PKC) activation [34]. Given that glucagon is one of several peptide hormones that increase glucose levels, Leibiger et al. investigated the broader implications of autocrine secretion–biosynthesis coupling, using glucagon as an example. Their study demonstrated that glucagon, through signaling via GCGR, PKC, and PKA, upregulates the expression of its own gene, providing evidence for a more widespread applicability of positive autocrine feedback [35].

Downstream signaling pathways of GCGRs involve the activation of Gs and Gq, leading to the formation of intracellular cAMP and inositol 1,4,5-trisphosphate (IP3), with the subsequent release of intracellular Ca^2+^. Activation of either Gs-induced protein kinase A (PKA) or Gq’s effect on Ca^2+^/calmodulin-dependent protein kinase can lead to the phosphorylation of the cAMP-response-element-binding protein (CREB). The metabolic effects of glucagon depend on its concentration, spatial features (mitochondrial vs. cytosolic effects), and substrate dependency. Hence, optimal plasma concentrations of substrates, specifically amino acids (AAs) and free fatty acids, play a pivotal role in both ureagenesis and gluconeogenesis processes. Prior investigations have elucidated that INSP3R1 is the isoform primarily responsible for mitochondrial calcium signaling in hepatocytes, and knocking down INSP3R1 reduced glucose production in isolated hepatocytes [36,37]. Perry et al. [29] implied that INSP3R1 activation and Ca^2+^/calmodulin-dependent protein kinase II (CAMKII) activity are important for the acute effect of glucagon in stimulating hepatic glucose production (HGP).

## 3. Glucagon and Glucose Metabolism

Glucose inhibits glucagon release upon oral or i.v. administration, while hypoglycemia increases the secretion of glucagon to elevate hepatic glucose output by stimulating glycogenolysis and gluconeogenesis, and it additionally inhibits glycogenesis and glycolysis and induces ketone production through multiple mechanisms, thereby protecting against hypoglycemia [27,38,39]. Glucagon acts in concert with cortisol, growth hormone, and adrenergic hormones, which also increase hepatic glucose output in hypoglycemia. Remarkably, the blockade of GCGRs does not impair the counter-regulation against hypoglycemia [7,40,41]. 

Although the level of glucagon rises rapidly in the early stage of fasting, circulating glucagon concentrations drop to postprandial levels upon prolonged fasting (see below), with persistently decreasing glycemia due to glycogen depletion [42]. Glucagon physiologically regulates the early phases of fasting in non-diabetic animals and humans, which is relevant in real life since prolonged fasting is an unusual state in industrialized societies. 

The inhibition of glucagon by increases in blood glucose is important to control blood glucose and is therefore finely tuned with insulin release. However, with the development of impaired glucose tolerance and T2DM, glucagon continues to induce glucose production during hyperglycemia in fasted and preprandial conditions [24,25,26]. Both fasting and postprandial hyperglucagonemia have been proposed to trigger metabolic disturbances in obese and/or prediabetic subjects [43,44]. In the 1970s, Unger et al. proposed that T2DM may not only be the consequence of relative or absolute insulin deficiency but also of glucagon excess in dogs [45]. Reaven et al. later showed that hyperglucagonemia persists throughout the day in both obese and non-obese patients with T2DM, despite significant elevations in plasma insulin and glucose levels. These factors are traditionally expected to inhibit glucagon secretion under normal conditions [46]. Recent studies have confirmed that most patients with T2DM exhibit abnormally high fasting plasma glucagon levels that do not appropriately decrease and, in some cases, may even increase after an oral glucose tolerance test (OGTT) or carbohydrate intake [24,26,47,48,49]. This failure to suppress glucagon secretion significantly contributes to postprandial hyperglycemia by increasing hepatic glucose production in T2DM patients. Some researchers suggest that the difficulty in controlling glucagon could signal an initial problem with α-cells in the pancreas before insulin deficiency develops. This might involve a reduced α-cell response to glucose and/or a lack of insulin’s suppressive effect within the pancreatic islets [21,43,50,51]. However, i.v. administration of glucose was reported to inhibit glucagon release similarly in people with or without diabetes. Since manifest diabetes is associated with increased fasting plasma glucose in the presence of increased plasma glucagon, the inhibition of glucagon release is partially lost. However, as further increases of glucose by i.v. infusion still inhibit glucagon release, the response appears to be maintained but right-shifted [52].

### 3.1. What Are the Explanations for This Difference (Oral vs. i.v. Glucose)?

A meticulous comparison of oral or i.v. glucose administration in healthy individuals revealed a more pronounced suppression of glucagon by i.v. compared with oral glucose [53]. Given that i.v. glucose lacks the capacity to elicit incretin responses, this cannot be explained by suppression of glucagon by GLP-1. However, as GIP was stimulated by oral but not i.v. glucose, one might postulate that GIP stimulated glucagon and thereby attenuated the effect of glucose, possibly due to the glucagonotropic action of GLP-2 [53]. In people with T2DM, an i.v. glucose dose dependently suppressed glucagon even at elevated basal plasma glucose levels, while oral glucose caused an initial stimulation of glucagon that was not explained by the levels of incretin hormones [54,55]. Complementary evidence has arisen from studies conducted on isolated α-cells and pancreatic islets. Notably, glucose unexpectedly stimulates glucagon release in isolated α-cells via mechanisms which involve K_ATP_ channels and Ca^2+^-mediated depolarization [56], highlighting potential indirect and paracrine mechanisms governing glucagon release inhibition in vivo. In addition, glucose inhibits glucagon release in intact mouse and human islets, apparently by paracrine mechanisms involving somatostatin release that have lost their function in islets from diabetic patients [57,58,59]. This would suggest that the deficient stimulation of somatostatin release by glucose or insulin from delta-cells in islets accounts for the hyperglucagonemia in diabetic conditions. Meanwhile, alternative somatostatin-independent mechanisms have also been proposed [59,60,61]. Recently, hyperglycemia-induced Na^+^-dependent reduction of ATP production and, thus mitochondrial impairment, was proposed as a primary mechanism [62,63]. One very recent study stated that glucagon secretion is inhibited by glucose through the involvement of pyruvate dehydrogenase and carnitine palmitoyl transferase 1a activity. This is accompanied by reduced mitochondrial fatty acid oxidation due to increased glucose levels, leading to lower intracellular ATP [64].

An important observation is that hyperglucagonemia in the fasting state and in response to arginine disappears upon prolonged infusion of insulin in insulin-dependent patients. This is compatible with a dysregulation of α-cells due to hyperglycemia and/or insulin deficiency and is not a primary defect of α-cell function [62,65]. This observation would suggest that glucose-induced dysregulation of energy metabolism in islets drives hyperglucagonemia. This raises the question of whether α-cell dysfunction also involves an exaggerated response to AAs or fat, which may also involve mitochondrial mechanisms. 

### 3.2. How Does Hypoglycemia Stimulate Glucagon Secretion?

Normally, hypoglycemia triggers a counter-regulatory response in the α-cells which does not happen in many T1DM and some T2DM patients. The comprehensive mechanisms by which glucose regulates glucagon secretion remain unclear. It has been claimed that CNS and hepatoportal sensors (i.e., hypoglycemia-activated gastrointestinal neurons in the brainstem and in several hypothalamic nuclei) contribute to the control of glucagon [66,67]. Recent studies also questioned whether the primary role of glucagon is solely to elevate glucose concentrations [68]. During the onset of fasting, there is an initial surge in glucagon levels along with a decrease in blood glucose. In prolonged fasts exceeding three days, a gradual reduction in circulating glucagon levels emerges. Surprisingly, these levels eventually normalize to what is typically seen after a meal, even in the presence of consistently low blood glucose [42]. Furthermore, the administration of glucagon in hypoglycemic individuals who have been fasting for more than three days does not produce any significant changes in glycemia, possibly due to depleted glycogen stores [69]. 

Two studies claimed that Irak4 (Interleukin-1 receptor associated kinase-4) controls hypoglycemia-induced glucagon secretion by modulating hypothalamic Il-1β signaling, and Agpat5 activation in AgRP (agouti-related protein) neurons leads to hypoglycemia-induced glucagon secretion [70,71]. A recent study uncovered a new function of liver Hnf4a (hepatocyte nuclear factor 4α) in regulating blood glucose levels through the control of glucagon signaling in mice [72]. It is established that sodium–glucose co-transporter 1 (SGLT-1) and the generation of reactive oxygen species (ROS) released from β-cells are involved in increasing α-cell proliferation and glucagon secretion [73]. The role of glucagon in contributing to hyperglycemia in diabetes was reinforced by observing lower glucose levels in mice whose expression of the glucagon receptors (GCGRs) has been knocked out [74], as well as by the improved glucose and HbA1c levels in humans treated with acute and prolonged pharmacological receptor antagonists (GRAs) in clinical and preclinical trials, although these were accompanied by hepatic side effects such as increases in transaminases, liver fat accumulation, and dyslipidemia in addition to α-cell hyperplasia [75,76,77]. 

The reason why counter-regulation fails in diabetic patients is not fully understood. Interestingly, inhibiting mitochondrial ATP production or pharmacologically activating K_ATP_ channels with diazoxide mimics the dysregulation of glucagon secretion [63]. These observations collectively suggest that the glucagon secretion defect in diabetic patients may stem from disrupted mitochondrial metabolism, though the exact mechanisms remain unclear.

It has been proposed that glucagon increases with the onset of obesity and fatty liver as a consequence of hepatic glucagon resistance [78] and with insulin resistance due to the inappropriate regulation of glucagon by fasting and a static glucagon/insulin ratio [79]. Normally, fasting induces an increase in the glucagon/insulin ratio, which leads to predominant glucagon signaling that increases hepatic intracellular concentrations of the second messenger cAMP and downstream PKA and CREB signaling pathways, increasing hepatic glucose output [80]. One study highlighted the role of nutrient signaling via mTOR complex 1 (mTORC1) regulation to control glucagon secretion and α-cell mass [81]. However, one recent study in mice demonstrated that chronic hyperglucagonemia can improve glucose homeostasis by downregulating hepatic GCGR expression, which induces hepatic “glucagon resistance”, and by enhancing insulin secretion [82].

## 4. Glucagon and Amino Acid Metabolism

In addition to its established glucose-regulatory effects, glucagon powerfully regulates hepatic AA turnover by increasing the activities of necessary transporters and enzymes in the urea cycle through cAMP–PKA–CREB signaling [83,84]. In fact, there is evidence to suggest that the impact of glucagon signaling may vary between fasting and postprandial conditions [85]. Glucagon activates the transcription of AA transporters located on the hepatocyte membrane, thus allowing increased AA uptake and substrate availability for ureagenesis [86]. In turn, AAs enhance glucagon secretion from α-cells [87]. This generates a glucagon and AA feedback loop, referred to as the “liver–α-cell axis”, which might be as important for metabolism as the glucagon–glucose loop [88,89,90,91,92]. Genetic interventions, GCGR-antibody or pharmacological inhibition of glucagon signaling leads to α-cell hypersecretion and hyperplasia as well as a decrease in the hepatic AA transporters and gene expression involved in AA metabolism, resulting in dramatically increased plasma concentrations of some but not all AAs [88,91,93]. Notably, decreased AA levels are linked to reduced target of rapamycin (mTOR) signaling in α-cells and suppressed α-cell proliferation [94]. Hormonally, protein-rich meals prompt glucagon secretion contingent on AA plasma level kinetics [95,96]. If excess AAs, i.e., more than can be utilized for protein synthesis, are taken up with meals or liberated by proteolysis upon fasting, they are used as energy substrates [97]. Since muscle and other organs lack the capacity to manage amino groups, leading to deamination of the AAs and the carbon moiety being taken for the Krebs cycle, and alanine is primarily used to shuttle the amino groups to the liver. Alanine therefore predominates within the 15 glucogenic AAs [98] and is preferentially taken up by the liver in the presence of elevated glucagon for glucose production (known as the glucose–alanine cycle or Cahill cycle) [99,100,101]. This cycle is dysregulated in dysglycemia in humans with obesity and T2DM, as exemplified by heightened splanchnic (that is, viscera and liver) alanine uptake. One recent study demonstrated that alanine transport and aminotransferase (ALT) isoform expression (ALT and ALT2) were remarkably higher in obese, prediabetes, and overtly diabetic mouse models and in individuals with metabolic diseases. In addition, a GCGR antagonist reduced hyperglycemia accompanied by blunting of the increased ALT/ALT2 activity in mice [100]. 

Glucagon’s influence extends beyond ureagenesis, encompassing renal nitrogen excretion [95]. Glucagon affects fluid and solute transport in the distal tubule and collecting duct by increasing hepatic cAMP secretion, which, in turn, influences the proximal tubule reabsorption of urea. This interaction increases the fractional excretion of urea, sodium, potassium, and phosphates. After oral protein loading, there was a significant correlation between GFR and the urinary urea nitrogen excretion rate [95]. Intriguingly, branched chain amino acids (BCAA) do not induce an increase in renal hemodynamics [102]. A postprandial increase in plasma glucagon could potentially counteract AA- and insulin-stimulated mTORC1 activation, leading to the suppression of protein synthesis in the liver. After ingesting a protein-rich diet, the liver shows increased rates of translation initiation and protein synthesis compared with fasted animals. A hypothesis posits that glucagon resistance, a molecular phenomenon impacting glucagon’s physiological effects on glucose, amino acid (AA), and lipid metabolism, might contribute to the development of T2DM and metabolic diseases [96]. In the healthy liver, glucagon binds to the hepatic GCGR and increases AA metabolism through urea production. Glucagon may influence several steps in this process, including various biochemical shunts in the carbamoyl phosphate and urea cycles. In subjects with liver diseases, such as NAFLD, GCGR resistance may affect this liver–α cell axis, as reduced hepatic GCGR expression or impaired GCGR signaling leads to decreased urea production, which, in turn, leads to hyperaminoacidemia and subsequent compensatory hyperglucagonemia. Furthermore, hepatic steatosis may impair the glucagon-dependent enhancement of AA catabolism in mice and humans by NAFLD [103]. A recent study reported that elevated plasma levels of total AAs are associated with hyperglucagonemia in NAFLD independently of glycemic control [104]. This hypothesis may explain the hyperglucagonemia frequently observed in obesity, fatty liver, and T2DM. However, the relative contributions of alpha- to beta-cell cross talk and diminished inhibition of glucagon secretion by insulin resistance or deficiency and increased AA stimulation is presently unclear. Both fatty liver and insulin resistance correlate well with glucagon levels in plasma [105].

Most circulating AAs have been shown to potently stimulate both glucagon and insulin secretion in animals and humans, albeit with varying effects among distinct AAs [106,107]. This augmented glucagon release is believed to prevent hypoglycemia after protein intake, as AAs also stimulate insulin secretion. As early as the 1970s, Unger had found that alanine infusion induced increased glucagon secretion with minimal impact on insulin in dogs. Lysine contributed to a lesser extent to α-cell secretion while BCAAs had no effects on glucagon secretion, whereas they elicited a significant insulin response [106,108,109,110]. Nevertheless, other studies have reported that BCAAs stimulate the secretion of both insulin and glucagon, particularly with oral administration, resulting in greater and more prolonged secretion of both hormones [111,112]. Later in 1974, arginine was proven to enhance both insulin and glucagon secretion, which support separate glucose and arginine receptors on both α- and β-cells in rodents, or directly promotes plasma membrane depolarization and Ca^2+^ influx in the α-cell [113,114]. In mice, leucine intake indeed improved glycemic control, while some studies observed negative effects on glucose homeostasis by methionine and BCAAs [115,116,117]. Furthermore, studies in animal- and human-isolated α-cells indicated that L-glutamine is a positive modulator of glucagon release and could regulate α-cell proliferation and mass via mTOR-dependent nutrient sensing [118,119]. In the following decades, clinical studies often utilized specific AAs, although the effects of individual AAs on glucagon secretion remain an area of controversy. Primarily, arginine is established as an α-cell secretagogue, which induces significant increases in circulating glucagon and insulin regardless of ambient glycemia [9]. Recent research demonstrated that BCAAs directly raised intracellular Ca^2+^ levels in α-cells, leading to increased plasma glucagon levels in diabetic mice. This suggests that disordered BCAA catabolism in pancreatic islet cells contributes to the postprandial hypersecretion of glucagon in diabetes [120]. Additionally, a separate study showed that glycine ingestion resulted in a slight decrease in serum glucose and increased insulin and glucagon concentrations in healthy subjects [121,122]. Galsgaard et al. proposed that defective glucagon signaling in the liver results in hyperaminoacidemia, which further stimulates the secretion of glucagon, possibly resulting in hyperplasia of the α-cells [123]. However, the precise number of AAs involved in the glucagon and AA feedback loop remains unclear, with glutamine suggested as a potential candidate [118]. Additionally, which AAs are capable of stimulating glucagon secretion directly from pancreatic α-cells or via increasing GCGR signaling remains mysterious. The Holst group also found that alanine, arginine, cysteine, and proline are involved in the acute regulation of the liver–α-cell axis through administering AA mixtures in vivo in mice [107]. Ingested whey protein in healthy participants induced hyperglucagonemia while suppressing free fatty acids. This demonstrates that physiological hyperglucagonemia can supersede the hepatic actions of insulin, and postprandial hyperglucagonemia collaborates synergistically with insulin to regulate glucose, amino acid, and nitrogen metabolism [124]. Epidemiological evidence indicates that plant-protein-based nutrition, which have lower methionine and BCAA and higher arginine content, is inversely associated with mortality and T2DM [125]. There are studies indicating that soy protein normalized fasting hyperglucagonemia and improved glucagon resistance in obese Zucker (fa/fa) rats through inducing increased GCGR recycling to the membrane of adipocytes and its ligand-binding and G-protein selectivity [125,126,127,128]. Our recent study showed that plant protein, with its lower methionine and BCAA but higher arginine content, leads to greater postprandial increases in glucagon compared with animal protein. As a result, it requires higher insulin levels to control glucose metabolism, which seems to be associated with the rate of AA appearance in patients with T2DM [129]. Moreover, our findings revealed that the glucagon responses to both whey and casein were moderately elevated in individuals with T2DM. This elevation occurred without a noticeable leftward shift of the dose–response curve. Notably, it was discerned that the impaired suppression of glucagon in the presence of glucose, coupled with heightened secretion in response to protein, was imperative to elicit the exaggerated glucagon response observed in this context [130].

## 5. Glucagon and Lipid Metabolism

Glucagon is also recognized for its potent hypolipidemic effects. In humans, intravenous glucagon administration reduces the amount of plasma cholesterol, total esterified fatty acids, and apolipoproteins and the hepatic synthesis of triglycerides by stimulating β-oxidation and lipolysis in the liver [131,132]. It has been shown that glucagon can modulate the expression and activity of peroxisome proliferator-activated receptors (PPARs), affecting various aspects of lipid metabolism [133]. Glucagon’s stimulation leads to the activation of PPARα, a subtype that plays a central role in fatty acid oxidation and lipid catabolism. This interaction enhances the breakdown of fatty acids and promotes their utilization as an energy source. Meanwhile, glucagon’s influence on PPARγ affects adipocyte differentiation and insulin sensitivity. This is crucial in the context of lipid metabolism as PPARγ controls genes related to adipogenesis and lipid storage. The interplay between glucagon and PPARs highlights the complex regulatory network that orchestrates lipid utilization and storage in response to varying metabolic demands. Furthermore, glucagon also reduces hepatic lipid accumulation and decreases hepatic lipid secretion through the inhibition of lipogenesis in the liver [134]. Glucagon inhibits the activity of acetyl-CoA carboxylase, a key enzyme that initiates fatty acid synthesis by converting acetyl-CoA to malonyl-CoA. This inhibition reduces the availability of malonyl-CoA, subsequently lowering fatty acid synthesis [135]. The impact of glucagon on cholesterol receptor expression in the liver primarily revolves around the LDL receptor (LDLR). The LDLR plays a key role in the uptake of low-density lipoprotein (LDL) particles, which carry cholesterol to various tissues, including the liver. Studies have shown that glucagon stimulation can lead to increased LDLR gene expression, enhancing the clearance of LDL particles from the bloodstream by the liver [75]. This contributes to the regulation of plasma cholesterol levels. Additionally, glucagon-induced activation of cAMP-responsive element-binding protein (CREB) can promote LDLR expression. The intricate interplay between glucagon, CREB, and LDLR demonstrates the hormone’s role in cholesterol homeostasis [75]. 

It is established that increased GCGR signaling has been linked to improved lipid metabolism. In 1979, studies explored glucagon’s role in the direct short-term regulation of hepatic free fatty acid (FFA) metabolism, which showed that physiological concentrations of glucagon increased ketogenesis and reduced triglyceride synthesis from palmitate in hepatocytes of rats fed at FFA concentrations of 1.0 mM or lower [136]. The intricate modulation of FFA metabolism by glucagon transpires through a dual mechanism involving both intrahepatic and extrahepatic pathways. Specifically, glucagon emerges as a suppressor of de novo fatty acid synthesis by inhibiting the formation of malonyl-CoA. This occurs after an increase in intracellular cAMP activates PKA, which phosphorylates and inactivates acetyl-CoA carboxylase (ACC) [137]. 

As GCGRs are expressed on β-cells and may stimulate insulin through both GLP-1R and GCGR, one may speculate that intra-islet regulation of insulin by glucagon might contribute to its effect on lipid metabolism. As discussed above, GCGR antagonists (e.g., LY2409021, Volagidemab) have been considered as glucose-lowering therapy in T2DM patients, but these resulted in lipid disorders, whereas glucagon/GLP-1 receptors co-agonism improved dyslipidemia and reduced hepatic steatosis, which have brought up discussions regarding to the relationship between glucagon signaling and lipid metabolism [5,138,139]. 

It remains unclear how glucagon promotes hepatic mitochondrial fat oxidation and to what extent glucagon influences lipolysis in adipose tissue, especially in humans. A previous study confirmed that INSP3R1 is essential due to the reduced glucose production seen when INSP3R1 expression was knocked down in isolated hepatocytes [37]. A recent groundbreaking discovery from Perry and co-workers [29] is quite impressive, who reported that glucagon stimulates intrahepatic lipolysis through INSP3R1/CAMKII-dependent activation with increased hepatic acetyl-CoA. In addition, glucagon stimulates hepatic mitochondrial oxidation through INSP3R1-mediated calcium signaling. The INSP3R1–ATGL pathway appears to play a central role in the regulation of hepatic lipid metabolism in response to glucagon. This is supported by the fact that plasma non-esterified fatty acid levels did not change significantly in both INSP3R1-LKO mice and the control mice during glucagon infusion [29]. Those results explain many of these actions since the cAMP/PKA-mediated effects were transcriptional and did not explain the acute metabolic actions of glucagon.

**Figure 2:**
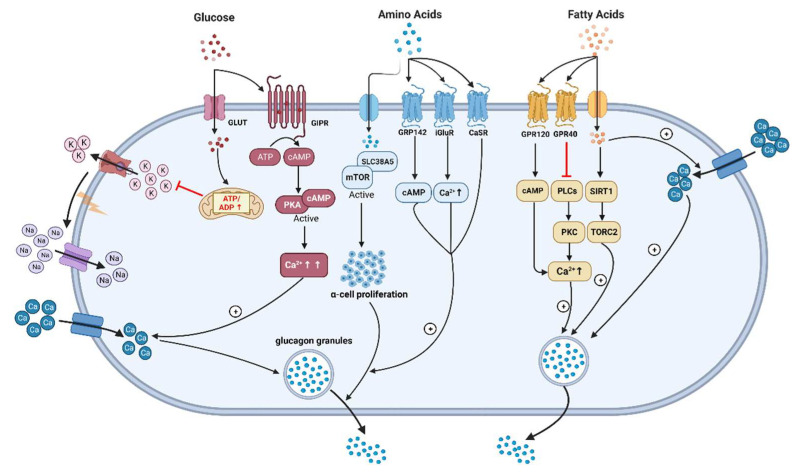
The underlying mechanisms of glucagon stimulation to glucose, amino acids, and fatty acids (↑increase). (This graph is generated with www.biorender.de).

In turn, the capability of FFAs to regulate glucagon secretion remains debatable, although they are insulin secretagogues under some circumstances and increased FFAs levels might be correlated with T2DM [21,140,141,142]. Early research in 1974 had shown that the elevation of plasma FFAs suppressed glucagon levels in people, which is supported by the following clinical studies [141,143,144]. Experiments carried out on isolated rodent islets, an α-cell line, and human islets have shown that FFAs (oleate or palmitate) stimulate glucagon secretion. This occurs through signaling via fatty acid G-protein-coupled receptors, the β-oxidation of fatty acids, and the activation of L-type Ca^2+^ channels. Additionally, it involves relieving the inhibitory paracrine action of somatostatin secreted from δ-cells [145,146,147]. Wang et al. reported that long-chain FFA (linoleic acid) acutely stimulated glucagon secretion by activation of G-protein-coupled receptor 40 (GPR40) and phospholipase C to increase Ca^2+^ release and the associated Ca^2+^ influx through Ca^2+^ channels in primary cultured rat pancreatic islets [148] (Figure 2). Similar effects have been observed in rat islets with oleic acid [149]. The Danish group reported that short-term exposure to FFAs directly increases glucagon release from α-cells. This stimulatory effect depends on the chain length, degree of unsaturation, and spatial configuration of FAs in both isolated mouse islets and alpha TC1-6 cells. Saturated fatty acids (SFA) were found to be more effective than unsaturated fatty acids (USFA) in stimulating glucagon secretion [146,150]. Subsequent data indicated that prolonged exposure (up to three days) to palmitate and oleate leads to excessive lipid accumulation and induces time- and dose-dependent hyperglucagonemia in both isolated islets and alpha TC1-6 cells through oxidation [151,152]. Long-term culture of a clonal α-cell line with palmitate increased glucagon release and expression, likely through activation of the AMPK pathway [153]. Conversely, insulin’s inhibitory effect on glucagon release was impaired after prolonged exposure to FFAs due to palmitate-induced insulin resistance, involving defects in the IRS-1/PI3K/Akt pathway [153]. In rat islets, chronic exposure to fatty acids resulted in increased glucagon release but decreased glucagon content without altering glucagon gene expression [154,155]. Glucolipotoxicity conditions, such as combining palmitate with high glucose levels, can induce apoptosis in rodent α-cells [156]. In clinical studies, intravenous or oral administration of a lipid emulsion did not alter glucagon secretion, and only oral lipid ingestion elicited a clear insulin response with increased GIP and GLP-1 concentrations [157]. In other studies, no difference in glucagon secretion was observed after a high-fat or low-fat diet intake in both never-obese and post-obese women [158]. However, an alternative study noted a slightly higher glucagon response to C4-dietary oil compared with C18-olive oil in overweight subjects with T2DM [159]. Furthermore, a sharp increase in plasma glucagon concentrations was observed in healthy men after the ingestion of long-chain fatty acids (olive oil and C8 fatty acids) while no increase in short-chain fatty acids (C4) was seen [160]. Another study reported that a 6-month intake of mono-unsaturated fatty acids (MUFAs) contributes to larger post-lunch glucagon responses compared with a control meal in healthy young subjects [161]. Interestingly, a recent study revealed that palmitate can cause a switch to a glucagon-secreting phenotype in intestinal GLP-1 secreting cells, suggesting the potential of fatty acids to induce extra-pancreatic glucagon [162]. Moreover, both long- and short-chain FFAs increase GIP concentrations, which might be one potential reason to stimulate glucagon release in humans [160,163]. Overall, the data from animals and humans support the hypothesis that the chronic elevation of fatty acids may contribute to α-cell deregulation in T2DM.

## 6. Conclusions

In summary, glucagon plays a crucial role in glucose, AA and lipid metabolism, with its secretion tightly regulated by glucose levels and other factors. In patients with impaired glucose tolerance and T2DM, dysregulation of glucagon secretion leads to elevated fasting plasma glucagon concentrations, even during hyperglycemia. The underlying mechanisms involve factors like incretins, somatostatin release, α-cell dysfunction, the loss of the crosstalk between pulsatile insulin and glucagon secretion, etc. The exact mechanisms driving these dysregulations are still under investigation, presenting potential avenues for therapeutic intervention in diabetes management.

Furthermore, the “liver–α-cell axis” links AAs to glucagon secretion, contributing to metabolic imbalances in conditions like obesity and T2DM. Specific AAs, such as arginine and BCAAS, directly influence plasma glucagon levels. Moreover, glucagon’s potent hypolipidemic effects are attributed to its role in hepatic β-oxidation, lipolysis, and inhibition of lipogenesis. However, the complex interplay between glucagon and fatty acids requires further elucidation to understand its implications in metabolic diseases. 

However, Kobayashi et al. recently revealed a significant issue with cross-reactivity in the conventional ELISA method, particularly in subjects with elevated plasma proglucagon-derived peptide levels [164]. This cross-reactivity might lead to incorrect results and, consequently, potential misinterpretation of blood glucagon levels from previous clinical data using traditional glucagon assays, which should be revisited and re-evaluated. 

Overall, investigating the intricate mechanisms governing glucagon’s diverse functions is vital to unraveling its role in metabolic disorders like T2DM. Advancing our understanding of glucagon’s regulatory pathways may offer new opportunities for developing targeted therapies to restore metabolic balance and improve patient outcomes.

## References

[1] Ricketts H.T. (1973). Glucagon: Molecular Physiology, Clinical and Therapeutic Implications. JAMA.

[2] Murlin J.R., Clough H.D., Gibbs C.B.F., Stokes A.M. (1923). Aqueous Extracts of Pancreas. J. Biol. Chem..

[3] Sutherland E.W., de Duve C. (1948). Origin and Distribution of the Hyperglycemic-Glycogenolytic Factor of the Pancreas. J. Biol. Chem..

[4] Sasaki H., Ebitani I., Tominaga M., Yamatani K., Yawata Y., Hara M. (1980). Glucagon-like substance in the canine brain. Endocrinol. Jpn..

[5] Guzman C.B., Zhang X.M., Liu R., Regev A., Shankar S., Garhyan P., Pillai S.G., Kazda C., Chalasani N., Hardy T.A. (2017). Treatment with LY2409021, a glucagon receptor antagonist, increases liver fat in patients with type 2 diabetes. Diabetes Obes. Metab..

[6] Pettus J.H., D’Alessio D., Frias J.P., Vajda E.G., Pipkin J.D., Rosenstock J., Williamson G., Zangmeister M.A., Zhi L., Marschke K.B. (2020). Efficacy and Safety of the Glucagon Receptor Antagonist RVT-1502 in Type 2 Diabetes Uncontrolled on Metformin Monotherapy: A 12-Week Dose-Ranging Study. Diabetes Care.

[7] Kazierad D.J., Chidsey K., Somayaji V.R., Bergman A.J., Calle R.A. (2018). Efficacy and safety of the glucagon receptor antagonist PF-06291874: A 12-week, randomized, dose-response study in patients with type 2 diabetes mellitus on background metformin therapy. Diabetes Obes. Metab..

[8] Bossart M., Wagner M., Elvert R., Evers A., Hubschle T., Kloeckener T., Lorenz K., Moessinger C., Eriksson O., Velikyan I. (2022). Effects on weight loss and glycemic control with SAR441255, a potent unimolecular peptide GLP-1/GIP/GCG receptor triagonist. Cell Metab..

[9] Finan B., Capozzi M.E., Campbell J.E. (2020). Repositioning Glucagon Action in the Physiology and Pharmacology of Diabetes. Diabetes.

[10] Rosenstock J., Wysham C., Frias J.P., Kaneko S., Lee C.J., Fernandez Lando L., Mao H., Cui X., Karanikas C.A., Thieu V.T. (2021). Efficacy and safety of a novel dual GIP and GLP-1 receptor agonist tirzepatide in patients with type 2 diabetes (SURPASS-1): A double-blind, randomised, phase 3 trial. Lancet.

[11] Sucher S., Markova M., Hornemann S., Pivovarova O., Rudovich N., Thomann R., Schneeweiss R., Rohn S., Pfeiffer A.F.H. (2017). Comparison of the effects of diets high in animal or plant protein on metabolic and cardiovascular markers in type 2 diabetes: A randomized clinical trial. Diabetes Obes. Metab..

[12] Markova M., Pivovarova O., Hornemann S., Sucher S., Frahnow T., Wegner K., Machann J., Petzke K.J., Hierholzer J., Lichtinghagen R. (2017). Isocaloric Diets High in Animal or Plant Protein Reduce Liver Fat and Inflammation in Individuals With Type 2 Diabetes. Gastroenterology.

[13] Unger R.H., Orci L. (2010). Paracrinology of islets and the paracrinopathy of diabetes. Proc. Natl. Acad. Sci. USA.

[14] Sherwin R., Felig P. (1977). Glucagon physiology in health and disease. Int. Rev. Physiol..

[15] Capozzi M.E., Wait J.B., Koech J., Gordon A.N., Coch R.W., Svendsen B., Finan B., D’Alessio D.A., Campbell J.E. (2019). Glucagon lowers glycemia when beta-cells are active. JCI Insight.

[16] Schulman J.L., Carleton J.L., Whitney G., Whitehorn J.C. (1957). Effect of glucagon on food intake and body weight in man. J. Appl. Physiol..

[17] Billington D.C. (1991). Angiogenesis and its inhibition: Potential new therapies in oncology and non-neoplastic diseases. Drug Des. Discov..

[18] Geary N., Kissileff H.R., Pi-Sunyer F.X., Hinton V. (1992). Individual, but not simultaneous, glucagon and cholecystokinin infusions inhibit feeding in men. Am. J. Physiol. Integr. Comp. Physiol..

[19] Bouscarel B., Kroll S.D., Fromm H. (1999). Signal transduction and hepatocellular bile acid transport: Cross talk between bile acids and second messengers. Gastroenterology.

[20] Holst J.J., Knop F.K., Vilsboll T., Krarup T., Madsbad S. (2011). Loss of incretin effect is a specific, important, and early characteristic of type 2 diabetes. Diabetes Care.

[21] Gromada J., Franklin I., Wollheim C.B. (2007). Alpha-cells of the endocrine pancreas: 35 years of research but the enigma remains. Endocr. Rev..

[22] Meier J.J., Nauck M.A., Pott A., Heinze K., Goetze O., Bulut K., Schmidt W.E., Gallwitz B., Holst J.J. (2006). Glucagon-like peptide 2 stimulates glucagon secretion, enhances lipid absorption, and inhibits gastric acid secretion in humans. Gastroenterology.

[23] Lang D.A., Matthews D.R., Burnett M., Ward G.M., Turner R.C. (1982). Pulsatile, synchronous basal insulin and glucagon secretion in man. Diabetes.

[24] Rohrer S., Menge B.A., Gruber L., Deacon C.F., Schmidt W.E., Veldhuis J.D., Holst J.J., Meier J.J. (2012). Impaired crosstalk between pulsatile insulin and glucagon secretion in prediabetic individuals. J. Clin. Endocrinol. Metab..

[25] Menge B.A., Gruber L., Jorgensen S.M., Deacon C.F., Schmidt W.E., Veldhuis J.D., Holst J.J., Meier J.J. (2011). Loss of inverse relationship between pulsatile insulin and glucagon secretion in patients with type 2 diabetes. Diabetes.

[26] Meier J.J., Kjems L.L., Veldhuis J.D., Lefebvre P., Butler P.C. (2006). Postprandial suppression of glucagon secretion depends on intact pulsatile insulin secretion: Further evidence for the intraislet insulin hypothesis. Diabetes.

[27] Habegger K.M., Heppner K.M., Geary N., Bartness T.J., DiMarchi R., Tschop M.H. (2010). The metabolic actions of glucagon revisited. Nat. Rev. Endocrinol..

[28] Ramnanan C.J., Edgerton D.S., Kraft G., Cherrington A.D. (2011). Physiologic action of glucagon on liver glucose metabolism. Diabetes Obes. Metab..

[29] Perry R.J., Zhang D., Guerra M.T., Brill A.L., Goedeke L., Nasiri A.R., Rabin-Court A., Wang Y., Peng L., Dufour S. (2020). Glucagon stimulates gluconeogenesis by INSP3R1-mediated hepatic lipolysis. Nature.

[30] Campbell J.E., Drucker D.J. (2013). Pharmacology, physiology, and mechanisms of incretin hormone action. Cell Metab..

[31] Bansal P., Wang Q. (2008). Insulin as a physiological modulator of glucagon secretion. Am. J. Physiol. Endocrinol. Metab..

[32] Oscar T.P. (1996). Down-regulation of glucagon receptors on the surface of broiler adipocytes. Poult. Sci..

[33] Kohout T.A., Lefkowitz R.J. (2003). Regulation of G protein-coupled receptor kinases and arrestins during receptor desensitization. Mol. Pharmacol..

[34] Ikegami T., Krilov L., Meng J., Patel B., Chapin-Kennedy K., Bouscarel B. (2006). Decreased glucagon responsiveness by bile acids: A role for protein kinase Calpha and glucagon receptor phosphorylation. Endocrinology.

[35] Leibiger B., Moede T., Muhandiramlage T.P., Kaiser D., Vaca Sanchez P., Leibiger I.B., Berggren P.O. (2012). Glucagon regulates its own synthesis by autocrine signaling. Proc. Natl. Acad. Sci. USA.

[36] Feriod C.N., Oliveira A.G., Guerra M.T., Nguyen L., Richards K.M., Jurczak M.J., Ruan H.B., Camporez J.P., Yang X., Shulman G.I. (2017). Hepatic Inositol 1,4,5 Trisphosphate Receptor Type 1 Mediates Fatty Liver. Hepatol. Commun..

[37] Wang Y., Li G., Goode J., Paz J.C., Ouyang K., Screaton R., Fischer W.H., Chen J., Tabas I., Montminy M. (2012). Inositol-1,4,5-trisphosphate receptor regulates hepatic gluconeogenesis in fasting and diabetes. Nature.

[38] Jiang G., Zhang B.B. (2003). Glucagon and regulation of glucose metabolism. Am. J. Physiol. Endocrinol. Metab..

[39] Sokal J.E. (1966). Effect of glucagon on gluconeogenesis by the isolated perfused rat liver. Endocrinology.

[40] Gerich J.E., Lorenzi M., Bier D.M., Tsalikian E., Schneider V., Karam J.H., Forsham P.H. (1976). Effects of physiologic levels of glucagon and growth hormone on human carbohydrate and lipid metabolism. Studies involving administration of exogenous hormone during suppression of endogenous hormone secretion with somatostatin. J. Clin. Investig..

[41] Lecavalier L., Bolli G., Gerich J. (1990). Glucagon-cortisol interactions on glucose turnover and lactate gluconeogenesis in normal humans. Am. J. Physiol..

[42] Marliss E.B., Aoki T.T., Unger R.H., Soeldner J.S., Cahill G.F. (1970). Glucagon levels and metabolic effects in fasting man. J. Clin. Investig..

[43] Demant M., Bagger J.I., Suppli M.P., Lund A., Gyldenlove M., Hansen K.B., Hare K.J., Christensen M., Sonne D.P., Holst J.J. (2018). Determinants of Fasting Hyperglucagonemia in Patients with Type 2 Diabetes and Nondiabetic Control Subjects. Metab. Syndr. Relat. Disord..

[44] Lee Y.H., Wang M.-Y., Yu X.-X., Unger R.H. (2016). Glucagon is the key factor in the development of diabetes. Diabetologia.

[45] Unger R.H., Orci L. (1975). The essential role of glucagon in the pathogenesis of diabetes mellitus. Lancet.

[46] Reaven G.M., Chen Y.D., Golay A., Swislocki A.L., Jaspan J.B. (1987). Documentation of hyperglucagonemia throughout the day in nonobese and obese patients with noninsulin-dependent diabetes mellitus. J. Clin. Endocrinol. Metab..

[47] Raskin P., Unger R.H. (1978). Hyperglucagonemia and its suppression. Importance in the metabolic control of diabetes. N. Engl. J. Med..

[48] Mitrakou A., Kelley D., Mokan M., Veneman T., Pangburn T., Reilly J., Gerich J. (1992). Role of reduced suppression of glucose production and diminished early insulin release in impaired glucose tolerance. N. Engl. J. Med..

[49] Ipp E. (2000). Impaired glucose tolerance: The irrepressible alpha-cell?. Diabetes Care.

[50] Sonne D.P., Rehfeld J.F., Holst J.J., Vilsboll T., Knop F.K. (2014). Postprandial gallbladder emptying in patients with type 2 diabetes: Potential implications for bile-induced secretion of glucagon-like peptide 1. Eur. J. Endocrinol..

[51] Shah P., Vella A., Basu A., Basu R., Schwenk W.F., Rizza R.A. (2000). Lack of suppression of glucagon contributes to postprandial hyperglycemia in subjects with type 2 diabetes mellitus. J. Clin. Endocrinol. Metab..

[52] Knop F.K., Vilsboll T., Madsbad S., Holst J.J., Krarup T. (2007). Inappropriate suppression of glucagon during OGTT but not during isoglycaemic i.v. glucose infusion contributes to the reduced incretin effect in type 2 diabetes mellitus. Diabetologia.

[53] Meier J.J., Deacon C.F., Schmidt W.E., Holst J.J., Nauck M.A. (2007). Suppression of glucagon secretion is lower after oral glucose administration than during intravenous glucose administration in human subjects. Diabetologia.

[54] Bagger J.I., Knop F.K., Lund A., Holst J.J., Vilsboll T. (2014). Glucagon responses to increasing oral loads of glucose and corresponding isoglycaemic intravenous glucose infusions in patients with type 2 diabetes and healthy individuals. Diabetologia.

[55] Bagger J.I., Knop F.K., Lund A., Vestergaard H., Holst J.J., Vilsboll T. (2011). Impaired regulation of the incretin effect in patients with type 2 diabetes. J. Clin. Endocrinol. Metab..

[56] Olsen H.L., Theander S., Bokvist K., Buschard K., Wollheim C.B., Gromada J. (2005). Glucose stimulates glucagon release in single rat alpha-cells by mechanisms that mirror the stimulus-secretion coupling in beta-cells. Endocrinology.

[57] Omar-Hmeadi M., Lund P.E., Gandasi N.R., Tengholm A., Barg S. (2020). Paracrine control of alpha-cell glucagon exocytosis is compromised in human type-2 diabetes. Nat. Commun..

[58] Kellard J.A., Rorsman N.J.G., Hill T.G., Armour S.L., van de Bunt M., Rorsman P., Knudsen J.G., Briant L.J.B. (2020). Reduced somatostatin signalling leads to hypersecretion of glucagon in mice fed a high-fat diet. Mol. Metab..

[59] Vergari E., Knudsen J.G., Ramracheya R., Salehi A., Zhang Q., Adam J., Asterholm I.W., Benrick A., Briant L.J.B., Chibalina M.V. (2019). Insulin inhibits glucagon release by SGLT2-induced stimulation of somatostatin secretion. Nat. Commun..

[60] Yu Q., Shuai H., Ahooghalandari P., Gylfe E., Tengholm A. (2019). Glucose controls glucagon secretion by directly modulating cAMP in alpha cells. Diabetologia.

[61] Almaca J., Molina J., Menegaz D., Pronin A.N., Tamayo A., Slepak V., Berggren P.O., Caicedo A. (2016). Human Beta Cells Produce and Release Serotonin to Inhibit Glucagon Secretion from Alpha Cells. Cell Rep..

[62] Knudsen J.G., Hamilton A., Ramracheya R., Tarasov A.I., Brereton M., Haythorne E., Chibalina M.V., Spegel P., Mulder H., Zhang Q. (2019). Dysregulation of Glucagon Secretion by Hyperglycemia-Induced Sodium-Dependent Reduction of ATP Production. Cell Metab..

[63] Zhang Q., Ramracheya R., Lahmann C., Tarasov A., Bengtsson M., Braha O., Braun M., Brereton M., Collins S., Galvanovskis J. (2013). Role of KATP channels in glucose-regulated glucagon secretion and impaired counterregulation in type 2 diabetes. Cell Metab..

[64] Armour S.L., Frueh A., Chibalina M.V., Dou H., Argemi-Muntadas L., Hamiltion A., Katzilieris-Petras G., Carmeliet P., Davies B., Moritz T. (2023). Glucose controls glucagon secretion by regulating fatty acid oxidation in pancreatic alpha cells. Diabetes.

[65] Gerich J.E., Tsalikian E., Lorenzi M., Schneider V., Bohannon N.V., Gustafson G., Karam J.H. (1975). Normalization of fasting hyperglucagonemia and excessive glucagon responses to intravenous arginine in human diabetes mellitus by prolonged infusion of insulin. J. Clin. Endocrinol. Metab..

[66] Steinbusch L., Labouebe G., Thorens B. (2015). Brain glucose sensing in homeostatic and hedonic regulation. Trends Endocrinol. Metab..

[67] Stanley S., Moheet A., Seaquist E.R. (2019). Central Mechanisms of Glucose Sensing and Counterregulation in Defense of Hypoglycemia. Endocr. Rev..

[68] Nauck M.A., Meier J.J. (2016). The incretin effect in healthy individuals and those with type 2 diabetes: Physiology, pathophysiology, and response to therapeutic interventions. Lancet Diabetes Endocrinol..

[69] Fisher M., Sherwin R.S., Hendler R., Felig P. (1976). Kinetics of glucagon in man: Effects of starvation. Proc. Natl. Acad. Sci. USA.

[70] Picard A., Berney X., Castillo-Armengol J., Tarussio D., Jan M., Sanchez-Archidona A.R., Croizier S., Thorens B. (2022). Hypothalamic Irak4 is a genetically controlled regulator of hypoglycemia-induced glucagon secretion. Mol. Metab..

[71] Strembitska A., Labouebe G., Picard A., Berney X.P., Tarussio D., Jan M., Thorens B. (2022). Lipid biosynthesis enzyme Agpat5 in AgRP-neurons is required for insulin-induced hypoglycemia sensing and glucagon secretion. Nat. Commun..

[72] Thymiakou E., Tzardi M., Kardassis D. (2023). Impaired hepatic glucose metabolism and liver-alpha-cell axis in mice with liver-specific ablation of the Hepatocyte Nuclear Factor 4alpha (Hnf4a) gene. Metabolism.

[73] Robson-Doucette C.A., Sultan S., Allister E.M., Wikstrom J.D., Koshkin V., Bhattacharjee A., Prentice K.J., Sereda S.B., Shirihai O.S., Wheeler M.B. (2011). Beta-cell uncoupling protein 2 regulates reactive oxygen species production, which influences both insulin and glucagon secretion. Diabetes.

[74] Johnson D.G., Goebel C.U., Hruby V.J., Bregman M.D., Trivedi D. (1982). Hyperglycemia of diabetic rats decreased by a glucagon receptor antagonist. Science.

[75] Spolitu S., Okamoto H., Dai W., Zadroga J.A., Wittchen E.S., Gromada J., Ozcan L. (2019). Hepatic Glucagon Signaling Regulates PCSK9 and Low-Density Lipoprotein Cholesterol. Circ. Res..

[76] Okamoto H., Cavino K., Na E., Krumm E., Kim S.Y., Cheng X., Murphy A.J., Yancopoulos G.D., Gromada J. (2017). Glucagon receptor inhibition normalizes blood glucose in severe insulin-resistant mice. Proc. Natl. Acad. Sci. USA.

[77] Okamoto H., Kim J., Aglione J., Lee J., Cavino K., Na E., Rafique A., Kim J.H., Harp J., Valenzuela D.M. (2015). Glucagon Receptor Blockade With a Human Antibody Normalizes Blood Glucose in Diabetic Mice and Monkeys. Endocrinology.

[78] Wewer Albrechtsen N.J., Pedersen J., Galsgaard K.D., Winther-Sorensen M., Suppli M.P., Janah L., Gromada J., Vilstrup H., Knop F.K., Holst J.J. (2019). The Liver-alpha-Cell Axis and Type 2 Diabetes. Endocr. Rev..

[79] Stern J.H., Smith G.I., Chen S., Unger R.H., Klein S., Scherer P.E. (2019). Obesity dysregulates fasting-induced changes in glucagon secretion. J. Endocrinol..

[80] Mutel E., Gautier-Stein A., Abdul-Wahed A., Amigo-Correig M., Zitoun C., Stefanutti A., Houberdon I., Tourette J.A., Mithieux G., Rajas F. (2011). Control of blood glucose in the absence of hepatic glucose production during prolonged fasting in mice: Induction of renal and intestinal gluconeogenesis by glucagon. Diabetes.

[81] Bozadjieva N., Blandino-Rosano M., Chase J., Dai X.Q., Cummings K., Gimeno J., Dean D., Powers A.C., Gittes G.K., Ruegg M.A. (2017). Loss of mTORC1 signaling alters pancreatic alpha cell mass and impairs glucagon secretion. J. Clin. Investig..

[82] Bozadjieva Kramer N., Lubaczeuski C., Blandino-Rosano M., Barker G., Gittes G.K., Caicedo A., Bernal-Mizrachi E. (2021). Glucagon Resistance and Decreased Susceptibility to Diabetes in a Model of Chronic Hyperglucagonemia. Diabetes.

[83] Snodgrass P.J., Lin R.C., Muller W.A., Aoki T.T. (1978). Induction of urea cycle enzymes of rat liver by glucagon. J. Biol. Chem..

[84] Hamberg O., Vilstrup H. (1994). Regulation of urea synthesis by glucose and glucagon in normal man. Clin. Nutr..

[85] Kazda C.M., Ding Y., Kelly R.P., Garhyan P., Shi C., Lim C.N., Fu H., Watson D.E., Lewin A.J., Landschulz W.H. (2016). Evaluation of Efficacy and Safety of the Glucagon Receptor Antagonist LY2409021 in Patients With Type 2 Diabetes: 12- and 24-Week Phase 2 Studies. Diabetes Care.

[86] Kilberg M.S., Barber E.F., Handlogten M.E. (1985). Characteristics and hormonal regulation of amino acid transport system A in isolated rat hepatocytes. Curr. Top. Cell Regul..

[87] Ohneda A., Parada E., Eisentraut A.M., Unger R.H. (1968). Characterization of response of circulating glucagon to intraduodenal and intravenous administration of amino acids. J. Clin. Investig..

[88] Dean E.D. (2020). A Primary Role for α-Cells as Amino Acid Sensors. Diabetes.

[89] Dean E.D., Unger R.H., Holland W.L. (2017). Glucagon antagonism in islet cell proliferation. Proc. Natl. Acad. Sci. USA.

[90] Flakoll P.J., Borel M.J., Wentzel L.S., Williams P.E., Lacy D.B., Abumrad N.N. (1994). The role of glucagon in the control of protein and amino acid metabolism in vivo. Metabolism.

[91] Boden G., Rezvani I., Owen O.E. (1984). Effects of glucagon on plasma amino acids. J. Clin. Investig..

[92] Holst J.J., Wewer Albrechtsen N.J., Pedersen J., Knop F.K. (2017). Glucagon and Amino Acids Are Linked in a Mutual Feedback Cycle: The Liver-alpha-Cell Axis. Diabetes.

[93] Miller R.A., Birnbaum M.J. (2016). Glucagon: Acute actions on hepatic metabolism. Diabetologia.

[94] Solloway M.J., Madjidi A., Gu C., Eastham-Anderson J., Clarke H.J., Kljavin N., Zavala-Solorio J., Kates L., Friedman B., Brauer M. (2015). Glucagon Couples Hepatic Amino Acid Catabolism to mTOR-Dependent Regulation of alpha-Cell Mass. Cell Rep..

[95] Bankir L., Bouby N., Speth R.C., Velho G., Crambert G. (2018). Glucagon revisited: Coordinated actions on the liver and kidney. Diabetes Res. Clin. Pr..

[96] Janah L., Kjeldsen S., Galsgaard K.D., Winther-Sorensen M., Stojanovska E., Pedersen J., Knop F.K., Holst J.J., Wewer Albrechtsen N.J. (2019). Glucagon Receptor Signaling and Glucagon Resistance. Int. J. Mol. Sci..

[97] Koopman R., Walrand S., Beelen M., Gijsen A.P., Kies A.K., Boirie Y., Saris W.H., van Loon L.J. (2009). Dietary protein digestion and absorption rates and the subsequent postprandial muscle protein synthetic response do not differ between young and elderly men. J. Nutr..

[98] Wahren J., Ekberg K. (2007). Splanchnic regulation of glucose production. Annu. Rev. Nutr..

[99] Felig P. (1973). The glucose-alanine cycle. Metabolism.

[100] Okun J.G., Rusu P.M., Chan A.Y., Wu Y., Yap Y.W., Sharkie T., Schumacher J., Schmidt K.V., Roberts-Thomson K.M., Russell R.D. (2021). Liver alanine catabolism promotes skeletal muscle atrophy and hyperglycaemia in type 2 diabetes. Nat. Metab..

[101] Snell K., Duff D.A. (1982). The hepato-muscular metabolic axis and gluconeogenesis. Prog. Clin. Biol. Res..

[102] Claris-Appiani A., Assael B.M., Tirelli A.S., Marra G., Cavanna G. (1988). Lack of glomerular hemodynamic stimulation after infusion of branched-chain amino acids. Kidney Int..

[103] Winther-Sorensen M., Galsgaard K.D., Santos A., Trammell S.A.J., Sulek K., Kuhre R.E., Pedersen J., Andersen D.B., Hassing A.S., Dall M. (2020). Glucagon acutely regulates hepatic amino acid catabolism and the effect may be disturbed by steatosis. Mol. Metab..

[104] Wewer Albrechtsen N.J., Junker A.E., Christensen M., Haedersdal S., Wibrand F., Lund A.M., Galsgaard K.D., Holst J.J., Knop F.K., Vilsboll T. (2018). Hyperglucagonemia correlates with plasma levels of non-branched-chain amino acids in patients with liver disease independent of type 2 diabetes. Am. J. Physiol. Gastrointest. Liver Physiol..

[105] Zhang J., Pivovarova-Ramich O., Kabisch S., Markova M., Hornemann S., Sucher S., Rohn S., Machann J., Pfeiffer A.F.H. (2022). High Protein Diets Improve Liver Fat and Insulin Sensitivity by Prandial but Not Fasting Glucagon Secretion in Type 2 Diabetes. Front. Nutr..

[106] Rocha D.M., Faloona G.R., Unger R.H. (1972). Glucagon-stimulating activity of 20 amino acids in dogs. J. Clin. Investig..

[107] Galsgaard K.D., Jepsen S.L., Kjeldsen S.A.S., Pedersen J., Wewer Albrechtsen N.J., Holst J.J. (2020). Alanine, arginine, cysteine, and proline, but not glutamine, are substrates for, and acute mediators of, the liver-alpha-cell axis in female mice. Am. J. Physiol. Endocrinol. Metab..

[108] Kaneto A., Kosaka K. (1972). Effects of leucine and isoleucine infused intrapancreatically on glucagon and insulin secretion. Endocrinology.

[109] Muller W.A., Faloona G.R., Unger R.H. (1971). The effect of alanine on glucagon secretion. J. Clin. Investig..

[110] Kuhara T., Ikeda S., Ohneda A., Sasaki Y. (1991). Effects of intravenous infusion of 17 amino acids on the secretion of GH, glucagon, and insulin in sheep. Am. J. Physiol..

[111] Gar C., Rottenkolber M., Prehn C., Adamski J., Seissler J., Lechner A. (2018). Serum and plasma amino acids as markers of prediabetes, insulin resistance, and incident diabetes. Crit. Rev. Clin. Lab. Sci..

[112] Adeva-Andany M.M., Lopez-Maside L., Donapetry-Garcia C., Fernandez-Fernandez C., Sixto-Leal C. (2017). Enzymes involved in branched-chain amino acid metabolism in humans. Amino Acids.

[113] Gerich J.E., Charles M.A., Grodsky G.M. (1974). Characterization of the effects of arginine and glucose on glucagon and insulin release from the perfused rat pancreas. J. Clin. Investig..

[114] Assan R., Attali J.R., Ballerio G., Boillot J., Girard J.R. (1977). Glucagon secretion induced by natural and artificial amino acids in the perfused rat pancreas. Diabetes.

[115] Zhang Y., Guo K., LeBlanc R.E., Loh D., Schwartz G.J., Yu Y.H. (2007). Increasing dietary leucine intake reduces diet-induced obesity and improves glucose and cholesterol metabolism in mice via multimechanisms. Diabetes.

[116] Newgard C.B., An J., Bain J.R., Muehlbauer M.J., Stevens R.D., Lien L.F., Haqq A.M., Shah S.H., Arlotto M., Slentz C.A. (2009). A branched-chain amino acid-related metabolic signature that differentiates obese and lean humans and contributes to insulin resistance. Cell Metab..

[117] Stone K.P., Wanders D., Orgeron M., Cortez C.C., Gettys T.W. (2014). Mechanisms of increased in vivo insulin sensitivity by dietary methionine restriction in mice. Diabetes.

[118] Dean E.D., Li M., Prasad N., Wisniewski S.N., Von Deylen A., Spaeth J., Maddison L., Botros A., Sedgeman L.R., Bozadjieva N. (2017). Interrupted Glucagon Signaling Reveals Hepatic alpha Cell Axis and Role for L-Glutamine in alpha Cell Proliferation. Cell Metab..

[119] Ostenson C.G., Grebing C. (1985). Evidence for metabolic regulation of pancreatic glucagon secretion by L-glutamine. Acta Endocrinol. (Copenh).

[120] Wada E., Kobayashi M., Kohno D., Kikuchi O., Suga T., Matsui S., Yokota-Hashimoto H., Honzawa N., Ikeuchi Y., Tsuneoka H. (2021). Disordered branched chain amino acid catabolism in pancreatic islets is associated with postprandial hypersecretion of glucagon in diabetic mice. J. Nutr. Biochem..

[121] Gannon M.C., Nuttall J.A., Nuttall F.Q. (2002). The metabolic response to ingested glycine. Am. J. Clin. Nutr..

[122] Kalogeropoulou D., LaFave L., Schweim K., Gannon M.C., Nuttall F.Q. (2009). Lysine ingestion markedly attenuates the glucose response to ingested glucose without a change in insulin response. Am. J. Clin. Nutr..

[123] Galsgaard K.D., Winther-Sørensen M., Ørskov C., Kissow H., Poulsen S.S., Vilstrup H., Prehn C., Adamski J., Jepsen S.L., Hartmann B. (2018). Disruption of glucagon receptor signaling causes hyperaminoacidemia exposing a possible liver-alpha-cell axis. Am. J. Physiol.-Endocrinol. Metab..

[124] Ang T., Bruce C.R., Kowalski G.M. (2019). Postprandial Aminogenic Insulin and Glucagon Secretion Can Stimulate Glucose Flux in Humans. Diabetes.

[125] Song M., Fung T.T., Hu F.B., Willett W.C., Longo V.D., Chan A.T., Giovannucci E.L. (2016). Association of Animal and Plant Protein Intake With All-Cause and Cause-Specific Mortality. JAMA Intern. Med..

[126] Diaz-Villasenor A., Granados O., Gonzalez-Palacios B., Tovar-Palacio C., Torre-Villalvazo I., Olivares-Garcia V., Torres N., Tovar A.R. (2013). Differential modulation of the functionality of white adipose tissue of obese Zucker (fa/fa) rats by the type of protein and the amount and type of fat. J. Nutr. Biochem..

[127] Tonstad S., Stewart K., Oda K., Batech M., Herring R.P., Fraser G.E. (2013). Vegetarian diets and incidence of diabetes in the Adventist Health Study-2. Nutr. Metab. Cardiovasc. Dis..

[128] Pan A., Sun Q., Bernstein A.M., Manson J.E., Willett W.C., Hu F.B. (2013). Changes in red meat consumption and subsequent risk of type 2 diabetes mellitus: Three cohorts of US men and women. JAMA Intern. Med..

[129] Markova M., Hornemann S., Sucher S., Wegner K., Pivovarova O., Rudovich N., Thomann R., Schneeweiss R., Rohn S., Pfeiffer A.F.H. (2018). Rate of appearance of amino acids after a meal regulates insulin and glucagon secretion in patients with type 2 diabetes: A randomized clinical trial. Am. J. Clin. Nutr..

[130] Zhang J., Schafer S.M., Kabisch S., Csanalosi M., Schuppelius B., Kemper M., Markova M., Meyer N.M.T., Pivovarova-Ramich O., Keyhani-Nejad F. (2023). Implication of sugar, protein and incretins in excessive glucagon secretion in type 2 diabetes after mixed meals. Clin. Nutr..

[131] Pegorier J.P., Garcia-Garcia M.V., Prip-Buus C., Duee P.H., Kohl C., Girard J. (1989). Induction of ketogenesis and fatty acid oxidation by glucagon and cyclic AMP in cultured hepatocytes from rabbit fetuses. Evidence for a decreased sensitivity of carnitine palmitoyltransferase I to malonyl-CoA inhibition after glucagon or cyclic AMP treatment. Biochem. J..

[132] Guettet C., Mathe D., Riottot M., Lutton C. (1988). Effects of chronic glucagon administration on cholesterol and bile acid metabolism. Biochim. Biophys. Acta.

[133] Monsalve F.A., Pyarasani R.D., Delgado-Lopez F., Moore-Carrasco R. (2013). Peroxisome proliferator-activated receptor targets for the treatment of metabolic diseases. Mediat. Inflamm..

[134] Galsgaard K.D., Pedersen J., Knop F.K., Holst J.J., Wewer Albrechtsen N.J. (2019). Glucagon Receptor Signaling and Lipid Metabolism. Front. Physiol..

[135] Peng I.C., Chen Z., Sun W., Li Y.S., Marin T.L., Hsu P.H., Su M.I., Cui X., Pan S., Lytle C.Y. (2012). Glucagon regulates ACC activity in adipocytes through the CAMKKbeta/AMPK pathway. Am. J. Physiol. Endocrinol. Metab..

[136] Witters L.A., Trasko C.S. (1979). Regulation of hepatic free fatty acid metabolism by glucagon and insulin. Am. J. Physiol..

[137] Watkins P.A., Tarlow D.M., Lane M.D. (1977). Mechanism for acute control of fatty acid synthesis by glucagon and 3’:5’-cyclic AMP in the liver cell. Proc. Natl. Acad. Sci. USA.

[138] Day J.W., Ottaway N., Patterson J.T., Gelfanov V., Smiley D., Gidda J., Findeisen H., Bruemmer D., Drucker D.J., Chaudhary N. (2009). A new glucagon and GLP-1 co-agonist eliminates obesity in rodents. Nat. Chem. Biol..

[139] Pocai A., Carrington P.E., Adams J.R., Wright M., Eiermann G., Zhu L., Du X., Petrov A., Lassman M.E., Jiang G. (2009). Glucagon-like peptide 1/glucagon receptor dual agonism reverses obesity in mice. Diabetes.

[140] Boden G., Carnell L.H. (2003). Nutritional effects of fat on carbohydrate metabolism. Best. Pr. Res. Clin. Endocrinol. Metab..

[141] Gerich J.E., Langlois M., Schneider V., Karam J.H., Noacco C. (1974). Effects of alternations of plasma free fatty acid levels on pancreatic glucagon secretion in man. J. Clin. Investig..

[142] Boden G., Shulman G.I. (2002). Free fatty acids in obesity and type 2 diabetes: Defining their role in the development of insulin resistance and beta-cell dysfunction. Eur. J. Clin. Investig..

[143] Madison L.L., Seyffert W.A., Unger R.H., Barker B. (1968). Effect on plasma free fatty acids on plasma glucagon and serum insulin concentrations. Metabolism.

[144] Edwards J.C., Taylor K.W. (1970). Fatty acids and the release of glucagon from isolated guinea-pig islets of Langerhans incubated in vitro. Biochim. Biophys. Acta.

[145] Collins S.C., Salehi A., Eliasson L., Olofsson C.S., Rorsman P. (2008). Long-term exposure of mouse pancreatic islets to oleate or palmitate results in reduced glucose-induced somatostatin and oversecretion of glucagon. Diabetologia.

[146] Olofsson C.S., Salehi A., Gopel S.O., Holm C., Rorsman P. (2004). Palmitate stimulation of glucagon secretion in mouse pancreatic alpha-cells results from activation of L-type calcium channels and elevation of cytoplasmic calcium. Diabetes.

[147] Kristinsson H., Sargsyan E., Manell H., Smith D.M., Gopel S.O., Bergsten P. (2017). Basal hypersecretion of glucagon and insulin from palmitate-exposed human islets depends on FFAR1 but not decreased somatostatin secretion. Sci. Rep..

[148] Wang L., Zhao Y., Gui B., Fu R., Ma F., Yu J., Qu P., Dong L., Chen C. (2011). Acute stimulation of glucagon secretion by linoleic acid results from GPR40 activation and [Ca2+]i increase in pancreatic islet {alpha}-cells. J. Endocrinol..

[149] Fujiwara K., Maekawa F., Dezaki K., Nakata M., Yashiro T., Yada T. (2007). Oleic acid glucose-independently stimulates glucagon secretion by increasing cytoplasmic Ca^2+^ via endoplasmic reticulum Ca^2+^ release and Ca^2+^ influx in the rat islet alpha-cells. Endocrinology.

[150] Hong J., Abudula R., Chen J., Jeppesen P.B., Dyrskog S.E., Xiao J., Colombo M., Hermansen K. (2005). The short-term effect of fatty acids on glucagon secretion is influenced by their chain length, spatial configuration, and degree of unsaturation: Studies in vitro. Metabolism.

[151] Hong J., Chen L., Jeppesen P.B., Nordentoft I., Hermansen K. (2006). Stevioside counteracts the alpha-cell hypersecretion caused by long-term palmitate exposure. Am. J. Physiol. Endocrinol. Metab..

[152] Hong J., Jeppesen P.B., Nordentoft I., Hermansen K. (2007). Fatty acid-induced effect on glucagon secretion is mediated via fatty acid oxidation. Diabetes Metab. Res. Rev..

[153] Piro S., Maniscalchi E.T., Monello A., Pandini G., Mascali L.G., Rabuazzo A.M., Purrello F. (2010). Palmitate affects insulin receptor phosphorylation and intracellular insulin signal in a pancreatic alpha-cell line. Endocrinology.

[154] Dumonteil E., Magnan C., Ritz-Laser B., Ktorza A., Meda P., Philippe J. (2000). Glucose regulates proinsulin and prosomatostatin but not proglucagon messenger ribonucleic acid levels in rat pancreatic islets. Endocrinology.

[155] Gremlich S., Bonny C., Waeber G., Thorens B. (1997). Fatty acids decrease IDX-1 expression in rat pancreatic islets and reduce GLUT2, glucokinase, insulin, and somatostatin levels. J. Biol. Chem..

[156] Ellingsgaard H., Ehses J.A., Hammar E.B., Van Lommel L., Quintens R., Martens G., Kerr-Conte J., Pattou F., Berney T., Pipeleers D. (2008). Interleukin-6 regulates pancreatic alpha-cell mass expansion. Proc. Natl. Acad. Sci. USA.

[157] Lindgren O., Carr R.D., Deacon C.F., Holst J.J., Pacini G., Mari A., Ahren B. (2011). Incretin hormone and insulin responses to oral versus intravenous lipid administration in humans. J. Clin. Endocrinol. Metab..

[158] Raben A., Holst J.J., Madsen J., Astrup A. (2001). Diurnal metabolic profiles after 14 d of an ad libitum high-starch, high-sucrose, or high-fat diet in normal-weight never-obese and postobese women. Am. J. Clin. Nutr..

[159] Mandoe M.J., Hansen K.B., Windelov J.A., Knop F.K., Rehfeld J.F., Rosenkilde M.M., Holst J.J., Hansen H.S. (2018). Comparing olive oil and C4-dietary oil, a prodrug for the GPR119 agonist, 2-oleoyl glycerol, less energy intake of the latter is needed to stimulate incretin hormone secretion in overweight subjects with type 2 diabetes. Nutr. Diabetes.

[160] Mandoe M.J., Hansen K.B., Hartmann B., Rehfeld J.F., Holst J.J., Hansen H.S. (2015). The 2-monoacylglycerol moiety of dietary fat appears to be responsible for the fat-induced release of GLP-1 in humans. Am. J. Clin. Nutr..

[161] Sloth B., Due A., Larsen T.M., Holst J.J., Heding A., Astrup A. (2009). The effect of a high-MUFA, low-glycaemic index diet and a low-fat diet on appetite and glucose metabolism during a 6-month weight maintenance period. Br. J. Nutr..

[162] Filippello A., Urbano F., Di Mauro S., Scamporrino A., Di Pino A., Scicali R., Rabuazzo A.M., Purrello F., Piro S. (2018). Chronic Exposure to Palmitate Impairs Insulin Signaling in an Intestinal L-cell Line: A Possible Shift from GLP-1 to Glucagon Production. Int. J. Mol. Sci..

[163] Freeland K.R., Wilson C., Wolever T.M. (2010). Adaptation of colonic fermentation and glucagon-like peptide-1 secretion with increased wheat fibre intake for 1 year in hyperinsulinaemic human subjects. Br. J. Nutr..

[164] Kobayashi M., Maruyama N., Yamamoto Y., Togawa T., Ida T., Yoshida M., Miyazato M., Kitada M., Hayashi Y., Kashiwagi A. (2023). A newly developed glucagon sandwich ELISA is useful for more accurate glucagon evaluation than the currently used sandwich ELISA in subjects with elevated plasma proglucagon-derived peptide levels. J. Diabetes Investig..

